# Factors associated with caregiver adherence to mobile health interstage home monitoring in infants with single ventricle or biventricular shunt-dependent heart disease

**DOI:** 10.1017/S1047951122001822

**Published:** 2022-06-08

**Authors:** Sydney R. Jackson, Shahryar M. Chowdhury, Frances K. Woodard, Sinai C. Zyblewski

**Affiliations:** 1 College of Medicine, Medical University of South Carolina, Charleston, SC, USA; 2 Department of Pediatrics, Medical University of South Carolina, Charleston, SC, USA

**Keywords:** Single ventricle, home monitoring programme, interstage, mobile health, telehealth

## Abstract

Mobile health technology is an emerging tool in interstage home monitoring for infants with single ventricle heart disease or biventricular shunt-dependent defects. This study sought to describe adherence to mobile health monitoring and identify factors and outcomes associated with adherence to mobile health monitoring. This was a retrospective, single-institution study of infants who were followed in a mobile health-based interstage home monitoring programme between February 2016 and October 2020. The analysis included 105 infants and subjects were grouped by frequency of adherence to mobile health monitoring. Within the study cohort, 16 (15.2%) had 0% adherence, 25 (23.8%) had <50% adherence, and 64 (61.0%) had >50% adherence. The adherent groups had a higher percentage of infants who were male (p = 0.02), white race (p < 0.01), non-Hispanic or non-Latinx ethnicity (p < 0.01) and had mothers with primary English fluency (p < 0.01), married marital status (p < 0.01), and a prenatal diagnosis of faetal cardiac disease (p = 0.03). Adherent groups also had a higher percentage of infants with non-Medicaid primary insurance (p < 0.01) and residence in a neighbourhood with a higher median household income (p < 0.04). Frequency of adherence was not associated with interstage mortality, unplanned cardiac reinterventions, or hospital readmissions. Impact of mobile health interstage home monitoring on caregiver stress as well as use of multi-language, low literacy, affordable mobile health options for interstage home monitoring warrant further investigation.

Infants with single ventricle physiology are medically fragile patients with risk for haemodynamic compromise and thus require close surveillance between staged surgical palliations. Since the initial publication of a novel home monitoring strategy by Ghanayem and colleagues, interstage home monitoring of objective clinical parameters, such as daily weight, oxygen saturations, and enteral intake, has become a standard of care amongst paediatric heart programmes to minimise the morbidity and mortality of infants with single ventricle heart disease between palliative interventions.^1–5^ There is widespread evidence that interstage home monitoring, in conjunction with conventional outpatient management, improves surgical and growth outcomes in infants with hypoplastic left heart syndrome and related variants.^2,4,6,7^

Telehealth platforms and mobile health applications are being used with increased frequency at some centres in efforts to improve the efficiency of communication between caregivers and providers, provide real-time image and video information, address socio-economic disparities in interstage survival, alleviate parental stress, and improve outcomes.^8–10^ In February 2016, our institution’s interstage home monitoring programme transitioned from a traditional paper log to a mobile health system for the caregivers’ daily recording of oxygen saturation and weight measurements. The mobile health interstage home monitoring programme integrated the modalities of the Epic electronic health records system (Epic Systems Corporation, Verona, WI) and its associated MyChart mobile device application. The mobile health system provided instantaneous transfer of home monitoring data into the patient’s electronic medical record, efficient transfer of data to the interstage monitoring team, real-time secure video conferencing capabilities between an interstage monitoring team member and a patient and/or caregiver, and improved transparency and accessibility of interstage monitoring data for the entire heart centre team. However, it is unknown if mobile health technology is widely accepted amongst caregivers, reduces healthcare disparities, and is associated with improved outcomes during the interstage period.

The objectives of this study were to describe caregiver adherence to mobile health interstage home monitoring and to identify factors and outcomes associated with adherence to mobile health interstage home monitoring.

## Materials and methods

### Study design

This study was a retrospective chart review at a single-site tertiary children’s hospital. Study inclusion criteria were as follows: 1) infants with either single ventricle cardiac physiology or biventricular shunt dependent lesions; 2) survival to hospital discharge during the neonatal hospitalisation; 3) enrollment in our institution’s interstage home monitoring programme between September 1, 2016 to October 31, 2020; and 4) plans for a second cardiac surgery (i.e. cavopulmonary anastomosis, comprehensive stage II palliation, or complete biventricular repair) at our centre. Infants who were listed for heart transplantation during the interstage period or underwent their planned second cardiac surgery at a different centre were excluded from the study. This study was approved by the institutional review board at the Medical University of South Carolina.

### Data collection

Study data were collected from the electronic medical records and included detailed information related to patient demographics, maternal and infant perinatal course, birth characteristics, neonatal hospital course, hospital discharge disposition, hospital readmissions, mortality during the interstage period, growth and nutrition between birth and admission for the planned second cardiac surgery, and the hospital course during the planned second cardiac surgery. Subjects who identified two or more races on their demographic information were categorised as “other” for the purposes of this study. Neighbourhood rurality and poverty data were collected using publicly available 2019 United States Census information, which provided statistics for all states, counties, and cities/towns with a population of 5000 or more.^11^ For patients living in cities/towns with a population of less than 5000, the Data USA database was used to collect neighbourhood socio-economic data.^12^ The distance from the subject’s home and the surgical centre was determined by using the “Fastest Route” on Google Maps.^13^

Noncardiac procedures were defined as surgeries necessary for the infant’s growth and survival outside of the hospital (e.g. gastrostomy tube placement, repair of congenital diaphragmatic hernia, repair of gastrointestinal congenital malformation). Minor elective procedures routinely performed in the outpatient environment were excluded (e.g. circumcision). Delayed sternal closure was counted as part of the primary cardiac surgical procedure. Unplanned cardiac reinterventions were defined as any surgical or catheter-based intervention outside of the initial primary cardiac procedure, scheduled pre-stage II catheterisation, and planned second cardiac surgery.

### Interstage home monitoring programme

All infants with single ventricle cardiac anatomy (both hypoplastic left heart syndrome and non- hypoplastic left heart syndrome defects) and biventricular shunt-dependent defects (e.g. tetralogy of Fallot with pulmonary atresia) were enrolled in our center’s interstage home monitoring programme as part of standard clinical care. All infants were discharged to home with a pulse oximeter and infant weighing scale. The components of the interstage home monitoring programme included (1) standardisation of the neonatal discharge process; (2) standardisation of caregiver education before neonatal discharge including red flag thresholds for seeking medical evaluation; (3) daily home monitoring by caregivers, including daily oxygen saturation measurement and weight measurement; (4) weekly phone calls to caregivers by a dedicated interstage home monitoring nurse practitioner; (5) involvement of a registered dietitian and selective use of feeding logs; (6) weekly meetings of a multidisciplinary interstage home monitoring team comprised of a cardiologist, nurse practitioner, and dietitian to review patient data; (7) biweekly paediatric cardiology outpatient clinic visits with focused echocardiograms; (8) evaluation at the Medical University of South Carolina Cardiac Neurodevelopment Clinic at 3 months of age; (9) pre-stage II heart catheterisation at 4 months of age for infants with single ventricle anatomy; and (10) stage II surgical palliation at 5 months of age for infants with single ventricle anatomy who underwent either a Norwood palliation or aortopulmonary shunt during the neonatal hospitalisation.

Between November 2011 and August 2016, our centre’s interstage home monitoring programme utilised a traditional paper log system for the caregivers’ daily recording of pulse oximeter and weight measurements. The caregivers recorded measurements at home in a paper log and during the nurse practitioner’s weekly phone call, the caregiver read aloud the pulse oximeter and weight measurements which were then subsequently recorded in a team ring binder stored in the nurse practitioner’s office. The data stored in the team ring binder were reviewed weekly during the multidisciplinary interstage monitoring team meetings. In September 2016, our centre transitioned from a traditional paper log to a mobile health interstage home monitoring system. The daily caregiver recording of home pulse oximeter and weight measurements onto a paper log was discontinued; instead, electronic submission of data through MyChart was adopted. Prior to hospital discharge, the nurse practitioner placed an order in Epic for “interstage monitoring” which subsequently activated a flowsheet within the patient’s electronic medical record specifically built to be populated by measurements entered through MyChart by the caregivers in the home environment. The interstage monitoring platform within Epic included the capacity to set provider alerts for pulse oximeter and weight measurements outside a designated range. The nurse practitioner assisted caregivers in uploading the MyChart application to their mobile devices and obtaining proxy access to their infant’s medical chart. Caregivers practiced using their home monitoring equipment, obtaining oxygen saturation and weight measurements, and submitting data into MyChart for three consecutive days on the cardiac step-down unit prior to hospital discharge. After hospital discharge, the recommended frequency of home monitoring was daily submission of weight and pulse oximeter measurements through MyChart. The data transfer into the infant’s electronic medical record was instantaneous through cellular service. The interstage home monitoring flowsheet was viewable to any member of the healthcare team who had access to the Epic electronic medical record. Through MyChart, caregivers had the capability to securely share photos with the medical team and the interstage monitoring team had the capacity to perform video visits which were defined as real-time secure video conferencing between a health care provider and a patient and/or family caregiver. The additional photo and video modalities of mobile health interstage monitoring were utilised specifically for the purposes of evaluating incision healing, gastrostomy tube sites, infant’s overall general appearance, infant’s respiratory rate and effort, and reinforcing caregiver teaching related to formula mixing and medication administration. There was no additional cost to families for the use of mobile health interstage monitoring, as MyChart is a free application for Apple and Android devices. MyChart was available only in English during the study period. After the transition to a mobile health system, the other core components of the interstage home monitoring programme including the standardised parent education, discharge processes, weekly phone calls to the caregivers, weekly meetings of the multidisciplinary interstage home monitoring team and frequency of scheduled outpatient cardiology evaluations remained unchanged.

### Statistical analysis

Adherence groups were pre-defined as 0% adherence (no MyChart entries), low adherence (MyChart entries <50% of interstage days), and high adherence (MyChart entries >50% of interstage days). Descriptive characteristics were compared between the adherence groups. Comparisons were assessed using Chi-square and Fisher–Freeman–Halton Exact tests for categorical variables and Mann–Whitney U or Kruskal–Wallis tests for continuous variables as appropriate. Bonferroni adjusted post hoc tests were assessed for comparisons between >2 groups. Univariable and stepwise multivariable logistic regression were performed to determine associations between independent variables and adherence to mobile health interstage home monitoring. Variables with p values of less than 0.2 upon univariable analysis were included in the multivariable model. Cox regression analysis and Kaplan Meier curves were utilised to identify associations between outcomes of interest (mortality, readmission, etc.) and independent variables. We had 95% power to detect differences in demographic variables with a medium effect size (d = 0.5) between mobile health adherence groups assuming α = 0.05. For regression analyses, we had 80% power to detect independent variables that were associated with adherence to the mobile health interstage home monitoring with an odds ratio of 1.5 assuming α = 0.05. Cox regression analyses had 85% power to detect whether an independent variable confers a hazard ratio of 1.7 to meet the outcome of interest. A p-value < 0.05 was considered statistically significant. Statistical analysis was performed using SPSS v.27 (IBM, Armonk, New York).

## Results

A total of 105 infants were included in the analysis. Within the study cohort, 16 (15.2%) infants had 0% adherence, 25 (23.8%) had low adherence (<50% of interstage days), and 64 (61.0%) had high adherence (>50% of interstage days) to performing and submitting home pulse oximeter and weight measurements into MyChart. There were differences in socio-economic and demographic characteristics between the mobile health adherence groups (Table 1). The high adherence group had a higher percentage of infants who were male (p = 0.02), white race (p < 0.01), and non-Hispanic or non-Latinx ethnicity (p < 0.01). The high adherence group also had a higher percentage of mothers with primary English fluency (p < 0.01), married marital status (p < 0.01), and a prenatal diagnosis of faetal cardiac disease (p = 0.03). The high adherence group had a higher percentage of infants who had non-Medicaid primary insurance (p < 0.01) and lived in a neighbourhood with a higher median household income (p < 0.04).

There were differences between the mobile health adherence groups with respect to feeding modality at the time of initial hospital discharge and age at the time of the second planned cardiac surgery. The high adherence group had a higher percentage of infants who required exclusive tube feeding at the time of hospital discharge (p = 0.03) and tended to be younger in age at the time of the second planned cardiac surgery (p < 0.01). There were no significant differences between groups in hospital readmissions, unplanned cardiac reinterventions, or mortality during the interstage period. There were no significant differences in weight-for-age z-score at the time of the second planned cardiac surgery (Table 1).

In multivariable analyses, increased adherence to mobile health interstage home monitoring was associated with younger age (B = −1.249, p < 0.01), lower weight (B = −0.553, p < 0.01), and lower weight-for-age z-score (B = −0.279, p < 0.034) at the time of the second planned cardiac surgery. Adherence was not associated with hospital readmissions, unplanned cardiac interventions, or mortality during the interstage period.

## Discussion

Over one-third of the study cohort (39%) did not consistently adhere to mobile health interstage home monitoring. Adherence was associated with demographic and socio-economic factors specifically race, ethnicity, primary language fluency, and surrogate markers of economic security. Though marital status is not a direct indicator of socio-economic status, married status is likely correlated to better financial security.^14^ Socio-economic factors have been shown to be associated with early mortality after stage 1 palliation in infants with single right ventricle defects and our study suggests that these factors remain important through the interstage period.^15^ Specifically, our study findings are consistent with previous studies that have identified lower socio-economic status to be associated with lower healthcare utilisation.^14^

The proliferation of consumer-grade mobile devices and secure, cloud-based services have improved the feasibility of real-time analytics and notifications to healthcare teams.^8,10^ In result, telehealth platforms are being increasingly used at some centres for interstage surveillance through telephone or tablet-based applications.^8,10,16^ These technologies may help address socio-economic disparities in interstage survival and potentially alleviate some parental burden and stress.^8,10,16–17^ However, in our study cohort, disparities in race, ethnicity, and socio-economic factors were observed regarding adherence to mobile health technology use. These disparities may be related to factors such as language barriers, lower literacy, financial insecurity, and insufficient prenatal care that negatively impacted engagement with medical teams. We hypothesise that additional factors, including limited cellular service especially in rural communities, inconsistent access to cellular service secondary to economic insecurity, and parental stress levels or mental health issues could also potentially play a part in widening the mobile healthcare accessibility gap and warrant further investigation. Recent data support national-level disparities in internet access and the adoption of mobile health technology may not address this challenge nor alleviate burdens related to limited healthcare access.^18^

We did not observe differences in hospital readmission, unplanned cardiac interventions, and interstage mortality between the zero, low, and high adherence groups. This negative finding may have been confounded by our centre’s partnership with a statewide paediatric home hospice and palliative care organisation that enabled infants enrolled in our interstage home monitoring programme and residing in South Carolina to receive weekly home nursing visits. In the zero-adherence group, 92% of infants received home hospice and palliative care services which included weekly home nursing visits for physical exams and assessments, social work support, child life support, multilingual translation services, assistance with transportation for medical appointments, and communication with medical teams and durable medical equipment companies. This increased level of in-home family support may have prevented acute, life-threatening medical events in our most resource-poor patients and/or disincentivised caregivers from engaging in mobile health technology.

Although mobile health adherence was not associated with hospital readmissions, unplanned cardiac interventions, and interstage mortality, it was associated with age and weight at the time of the planned second cardiac surgery. Infants in the high mobile health adherence group were more likely to be younger and have lower absolute weight and weight-for-age z-score at the time of the planned second cardiac surgery. These findings merit further investigation, but we hypothesise that patients may have had an earlier surgical referral for feeding and growth problems because such symptoms suggested a sicker state. Additionally, caregivers with an infant exhibiting symptoms of compromised cardiac physiology may have been more motivated to adhere.

This study was limited by the single-centre, retrospective study design. The groups had small sample sizes and were heterogeneous in regard to cardiac anatomy. During the study period, our institution’s MyChart was available only in English; thus, adherence was biased towards English-speaking caregivers. United States Census data was unavailable for neighbourhood rurality and poverty data for cities/towns with populations of less than 5000; therefore, a different source had to be utilised for poverty data for study subjects living in less populated cities/towns. Programmatic changes to our institution’s interstage home monitoring programme during the study period that may have confounded the study results and outcomes include the integration of supportive paediatric home hospice and palliative care services starting in February 2018. Additionally, the adoption of routine use of digoxin to prevent interstage death in infants who have undergone a Norwood or hybrid procedure started in January 2019 and may have confounded mortality outcomes. Factors such as caregivers’ level of stress were not studied due to insufficient data.

In conclusion, in our study cohort, adherence to mobile health interstage home monitoring was associated with demographic and socio-economic factors specifically race, ethnicity, primary language fluency, and surrogate markers of economic security. However, adherence was not associated with differences in interstage mortality. Large, multi-institutional studies are warranted to study the acceptance and efficacy of telehealth or mobile health interstage home monitoring programmes amongst diverse populations. The impact of multilanguage, low literacy, affordable telehealth, and social media options for monitoring and communicating with families during the interstage period requires further investigation.

## Figures and Tables

**Table 1.** Comparison of patient characteristics grouped by adherence to mHealth.

	0% adherence (n = 16)	< 50% adherence (n = 25)	> 50% adherence (n = 64)	p-value
Male infant sex, n (%)	11 (68.8)	18 (72.0)	28 (43.8)	0.02 ^^^
Race				<0.01^^^*
White	5 (31.3)	7 (28.0)	45 (70.3)	
Black	5 (31.3)	15 (60.0)	13 (20.3)	
Other	6 (37.5)	3 (12.0)	6 (9.4)	
Hispanic/Latinx ethnicity	3 (18.8)	0 (0.0)	0 (0.0)	<0.01*
Primary language fluency				<0.01^#^*
English	11 (68.8)	24 (96.0)	64 (100.0)	
Spanish	4 (25)	0 (0.0)	0 (0.0)	
Other	1 (6.3)	1 (4.0)	0 (0.0)	
City/town population <5000	6 (37.5)	4 (16.0)	11 (17.2)	0.18
Married maternal marital status	5 (33.3)	8 (33.3)	47 (77.0)	<0.01^#^^
Medicaid insurance	13 (81.3)	18 (72.0)	23 (35.9)	<0.01^#^^
Neighbourhood median household income	37,062 (30,090, 46,264)	45,510 (38,199, 56,668)	51,065 (40,053, 65,375)	0.04*
Neighbourhood poverty rate	0.22 (0.21, 0.24)	0.19 (0.12, 0.23)	0.17 (0.09, 0.22)	0.08
Distance of home from surgical centre	122 (92, 209)	133 (56, 186)	123 (96, 209)	0.73
Prenatal diagnosis	10 (62.5)	23 (92.0)	55 (85.9)	0.03
Maternal age at birth	28 (23, 31)	27 (21, 32)	29 (25, 32)	0.21
Maternal gravida 1	5 (33.3)	8 (32.0)	23 (36.5)	0.89
Gestational age at birth				0.53
<37 weeks	2 (12.5)	5 (20.0)	6 (9.4)	
37 – 37 6/7 weeks	3 (18.8)	4 (16.0)	11 (17.2)	
38 – 38 6/7 weeks	3 (18.8)	4 (16.0)	10 (15.6)	
39 – 39 6/7 weeks	4 (25.0)	10 (40.0)	31 (48.4)	
40 – 40 6/7 weeks	3 (18.8)	2 (8.0)	6 (9.4)	
41+ weeks	1 (6.3)	0 (0.0)	0 (0.0)	
Weight-for-age z-score at birth	−0.34 (−0.74, 0.43)	−0.67 (−2.01, 0.24)	−0.11 (−1.04, 0.51)	0.33
Genetic or chromosome abnormality	2 (12.5)	5 (20.0)	9 (14.1)	0.74
Presence of extracardiac anomalies	8 (50.0)	12 (48.0)	18 (28.1)	0.10
Neonatal cardiac palliation type				0.20
Norwood	6 (37.5)	4 (16.0)	26 (40.6)	
Hybrid	2 (12.5)	2 (8.0)	7 (10.9)	
Shunt	3 (18.8)_a, b_	13 (52.0)_b_	16 (25.0)_a_	
Other	5 (31.3)	6 (24.0)	15 (23.5)	
Duration of initial hospitalisation, days	55 (41, 85)	42 (32, 70)	44 (33, 56)	0.16
Feeding modality at initial discharge				0.03 ^^^
Oral	8 (50.0)	17 (68.0)	22 (34.4)	
Oral/tube	1 (6.3)	0 (0.0)	11 (17.2)	
Tube	7 (43.8)	8 (32.0)	31 (48.4)	
Total number of medications at initial discharge	5 (3, 5)	4 (3, 4)	5 (3, 5)	0.14
Weight-for-age z-score at initial discharge	−2.36 (−2.78, −1.34)	−1.7 (−3.45, −0.69)	−1.47 (−2.06, −0.79)	0.23
Enrollment in home hospice or palliative care	12 (92.3)	17 (70.8)	37 (66.1)	0.18
Unplanned cardiac re-intervention during interstage	6 (37.5)	6 (24.0)	16 (25.0)	0.07
Hospital re-admission to surgical centre during interstage	1 (0, 2)	1 (1, 2)	1 (0, 2)	0.46
Interstage mortality	0 (0.0)	2 (8.0)	4 (6.3)	0.72
Age at time of 2^nd^ cardiac surgery, months	8 (4.8, 11.5)	8 (6, 12)	6 (5, 7)	<0.01^^^
Weight-for-age z-score at 2^nd^ cardiac surgery	−0.78 (−1.59, −0.04)	−1.74 (−2.59, −0.36)	−1.27 (−2.01, −0.51)	0.21
Duration of 2^nd^ cardiac surgery hospitalisation	14.5 (7, 20.3)	7 (6, 13)	9 (7, 20)	0.16

Continuous data reported as median (interquartile range). Categorical data reported as count (percentage).

# Statistically significant difference detected between 0% adherence and <50% adherence

^ Statistically significant difference detected between <50% adherence and ≥50% adherence

* Statistically significant difference detected between 0% adherence and ≥50% adherence
